# Phosphoinositide lipids in primary cilia biology

**DOI:** 10.1042/BCJ20200277

**Published:** 2020-09-24

**Authors:** Sarah E. Conduit, Bart Vanhaesebroeck

**Affiliations:** UCL Cancer Institute, University College London, 72 Huntley Street, London WC1E 6BT, U.K.

**Keywords:** ciliopathy, phosphoinositides, primary cilia

## Abstract

Primary cilia are solitary signalling organelles projecting from the surface of most cell types. Although the ciliary membrane is continuous with the plasma membrane it exhibits a unique phospholipid composition, a feature essential for normal cilia formation and function. Recent studies have illustrated that distinct phosphoinositide lipid species localise to specific cilia subdomains, and have begun to build a ‘phosphoinositide map’ of the cilium. The abundance and localisation of phosphoinositides are tightly regulated by the opposing actions of lipid kinases and lipid phosphatases that have also been recently discovered at cilia. The critical role of phosphoinositides in cilia biology is highlighted by the devastating consequences of genetic defects in cilia-associated phosphoinositide regulatory enzymes leading to ciliopathy phenotypes in humans and experimental mouse and zebrafish models. Here we provide a general introduction to primary cilia and the roles phosphoinositides play in cilia biology. In addition to increasing our understanding of fundamental cilia biology, this rapidly expanding field may inform novel approaches to treat ciliopathy syndromes caused by deregulated phosphoinositide metabolism.

## Introduction

**Figure 1.**
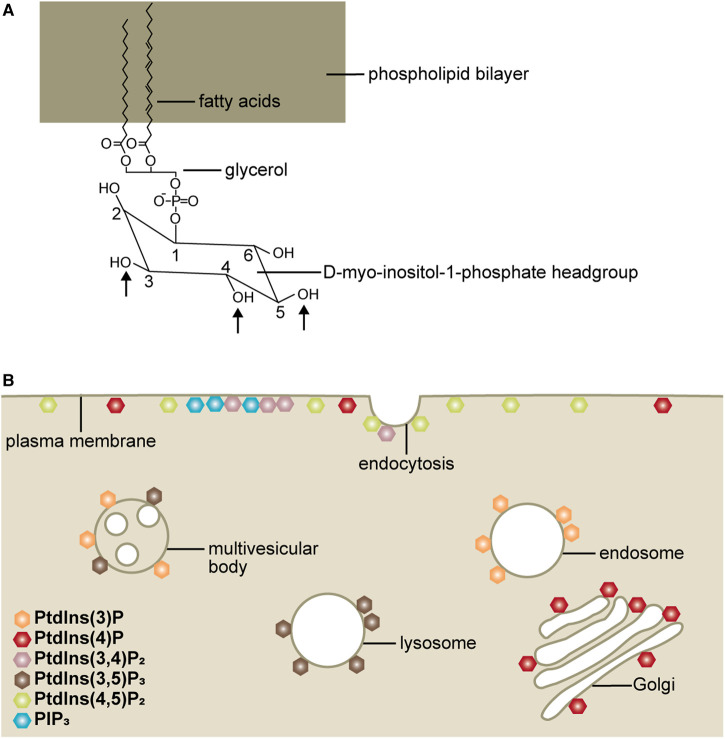
Localisation of phosphoinositides defines membrane identity. (**A**) Structure of PtdIns, arrows highlight the 3-, 4- and 5-positions of the inositol ring which can be phosphorylated. (**B**) Distinct phosphoinositides localise to the cytoplasmic leaflet of specific membrane domains and thereby confer membrane identity and control trafficking and signalling events. The major phosphoinositides at the membrane domains are depicted. PtdIns(4,5)P_2_ is the major species at the plasma membrane, although lower levels of PtdIns(4)P are also detected and PIP_3_ and PtdIns(3,4)P_2_ are transiently produced following growth factor stimulation. The endocytic membranes are predominantly decorated by PtdIns(3)P which is converted to PtdIns(3,5)P_2_ as vesicles progress through the endocytic network to the multivesicular body and lysosome. PtdIns(4)P is the major Golgi-resident phosphoinositide.

Phosphoinositides are minor membrane lipids that regulate critical cell biological processes, largely by dictating protein localisation and activity. Phosphoinositides are built around the phosphatidylinositol (PtdIns) building block (Figure 1A) by phosphorylation at different sites of the inositol ring. This gives rise to multiple species of phosphoinositides which show a characteristic distribution over different compartments of the cell, including the plasma membrane and internal membranous organelles, thereby defining membrane identity (Figure 1B) [1–3]. Phosphoinositides have been extensively studied for the last 50 years, however, only recently has a previously unappreciated subcellular localisation and function for these lipids emerged at the primary cilium [4–10].

The importance of phosphoinositides in regulation of primary cilia was first recognised in 2009 when seminal studies by Jacoby et al. [11] and Bielas et al. [12] identified that mutations in the inositol polyphosphate 5-phosphatase *INPP5E* are causally implicated in two ciliopathy syndromes. However, it took another six years until the first phosphoinositides [PtdIns(4)P and PtdIns(4,5)P_2_] were shown to localise to the ciliary membrane [4,5].

Collectively, PtdIns(4)P, PtdIns(4,5)P_2_, PtdIns(3,4,5)P_3_ (also known as PIP_3_) and PtdIns(3,4)P_2_ have since been identified in cilia membrane subdomains, with PtdIns(3)P found at a cilia-associated endosomal compartment and PtdIns(4)P detected at the centrosome prior to cilia assembly [4–10]. This review will explore the localisation and function of these phosphoinositides and their regulatory enzymes in primary cilia subdomains. We will begin by defining the structure of the primary cilium, which is compartmentalised into domains with distinct phosphoinositide compositions and describe the critical role of cilia in cell and developmental biology. Phosphoinositides will then be introduced and their emerging roles in cilia biology and the effects of mutation or deletion of the ciliary lipid kinases and phosphatases discussed. For more details on phosphoinositide function and regulation outside the cilium the reader is referred to the following excellent recent reviews [1–3,13–15].

## Primary cilia

### Primary cilia structure

Primary cilia are microtubule-based organelles projecting from the surface of most non-dividing cells that concentrate signalling molecules and are critical for transduction of multiple signalling pathways [16]. A cell expresses a single primary cilium. In contrast, specialised cell types often exhibit multiple motile (secondary) cilia, which actively move to generate water movement, resulting in fluid flow or cell motility. Phosphoinositides have not been examined in the context of motile cilia, this review will therefore focus on the role and localisation of phosphoinositides at primary cilia.

**Figure 2.**
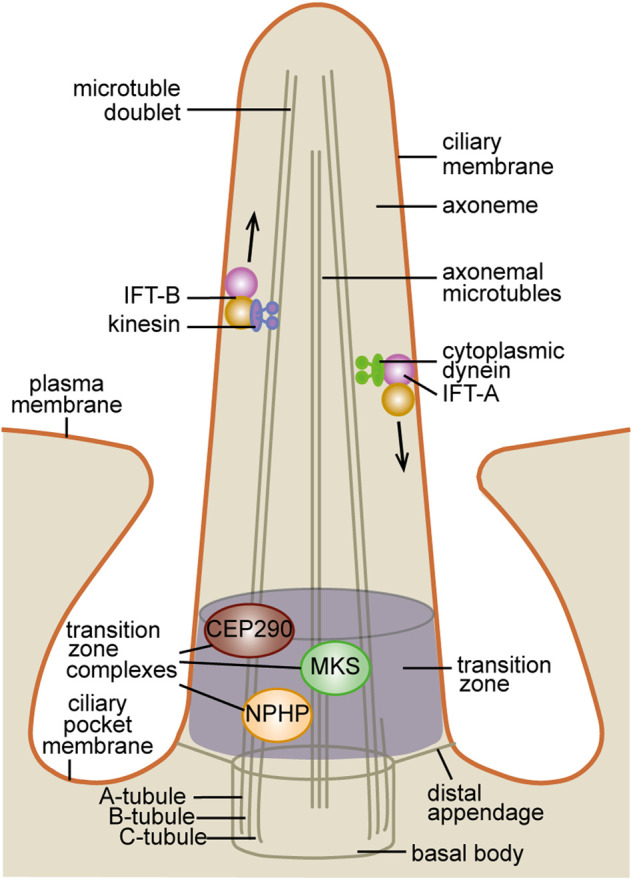
Primary cilia structure. The primary cilium is built around a scaffold of microtubules known as the axoneme. The microtubules that make up the axoneme are arranged as nine doublets (each doublet contains an A tubule and a B tubule). At the base, the axoneme is anchored by the basal body. The basal body is a modified mother centriole which has acquired distal appendage proteins that it uses to dock with the plasma membrane. It consists of nine microtubule triplets (A–C tubules). The axoneme is covered by the ciliary membrane, a membrane domain continuous with the plasma membrane but separated by a diffusion barrier. The ciliary membrane originates from Golgi-derived pre-ciliary vesicles and exhibits a unique lipid and protein composition relative to the plasma membrane. The membrane domain between the ciliary membrane and the plasma membrane is known as the ciliary pocket and is proposed to be the site where cargo from the endocytic network enter the ciliary membrane. A diffusion barrier at proximal end of the axoneme allows the cilium to maintain autonomy from the rest of the cytoplasm. The diffusion barrier is made up of three multi-protein complexes (MKS, NPHP and CEP290) at the transition zone, phosphoinositide binding Septins and the basal body distal appendages proteins (also known as transition fibres). The primary cilium does not synthesise its own protein components, therefore all proteins must be trafficked into and within the cilium. The IFT system mediates trafficking within the cilium. Kinesin microtubule motors walk along the axonemal microtubules and mediate anterograde IFT in complex with the IFT-B proteins which bind cargo molecules. Conversely, retrograde IFT is mediated by the microtubule motor cytoplasmic dynein and the IFT-A complex.

The primary cilium consists of the so-called axoneme made up of microtubules arranged as nine doublets (9 + 0, as opposed to the 9 + 2 microtubule arrangement of motile cilia which have an additional central pair) anchored by a modified mother centriole, termed the basal body (Figure 2). The ciliary membrane ensheaths the axoneme and although continuous with the plasma membrane, it is enriched with specific proteins and phospholipids. Indeed, as explained below, the ciliary membrane is derived from an endomembrane not the plasma membrane.

The region at the cilia base between the axoneme and basal body is defined as the transition zone and is made up of the MKS, NPHP and CEP290 multi-protein complexes (reviewed in [17]). These complexes act as a diffusion barrier that controls the entry and retention of membrane and soluble molecules [18,19]. The transition zone complexes are made up of multiple transmembrane, membrane-associated and soluble proteins that depend on one another for ciliary localisation [18,19]. Interestingly, many transition zone proteins such as AHI1, NPHP4, NPHP1, NPHP8, CC2D2A and RPGRIP1L contain phosphoinositide binding domains [18,20] but the identity of their lipid binding partners *in vivo* and the significance of these interactions are unclear. Mutations in transition zone proteins lead to major defects in primary cilia function such as disruption of assembly or failure of ciliary proteins to concentrate within the axoneme [18,19].

**Figure 3.**
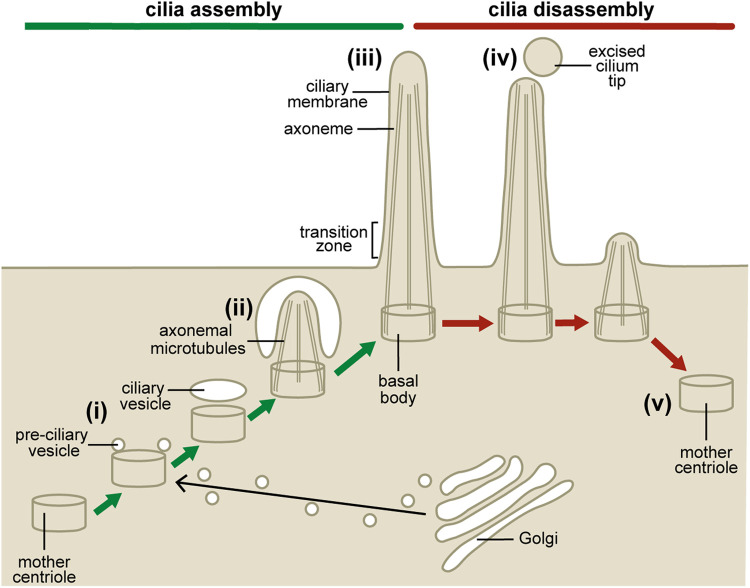
Primary cilia dynamics. Primary cilia assembly occurs when cells exit the cell cycle or in response to developmental signals. (**i**) Golgi-derived pre-ciliary vesicles dock with the mother centriole and fuse to become the ciliary vesicle. The mother centriole differentiates into the basal body by the acquisition of accessory structures and microtubule capping protein CP110 is removed to enable microtubule elongation. (**ii**) A nascent cilium is formed within the cytosol by microtubule assembly from the basal body which is covered by the double membrane ciliary vesicle. (**iii**) Finally the ciliary vesicle fuses with the plasma membrane to reveal the mature cilium projecting into the extracellular space. (**iv**) Primary cilia disassemble as cells progress through the cell cycle in response to growth factor stimulation. Disassembly occurs via microtubule destabilisation and disassembly. Cilia decapitation also contributes to disassembly by removing positive regulators of cilia maintenance. (**v**) Once the cilium has disassembled the mother centriole is released for mitotic spindle formation.

*In vitro* primary cilia assemble in a process also known as ciliogenesis (Figure 3(i–iii)) when cells exit the cell cycle, with cilia resorbed following growth factor stimulation (cilia disassembly; Figure 3(iv,v)), releasing the mother centriole to function as an anchor for the mitotic spindle [21]. Removal of serum from cell culture media is a well-established *in vitro* procedure to induce ciliogenesis. The *in vivo* initiating signal(s) is/are less well established but are thought to be via the induction of a ciliary transcriptional network promoting cilia gene expression [22].

Upon initiation of ciliogenesis, Golgi-derived pre-ciliary vesicles are recruited to and dock with the mother centriole [23]. The pre-ciliary vesicles fuse via SNARE protein interactions to form the ciliary vesicle, the mother centriole acquires distal appendage proteins to differentiate into the basal body and the transition zone complexes are recruited [24]. Microtubules assemble from the basal body and extension of the overlying ciliary vesicle occurs via fusion of additional vesicles, resulting in the formation of a nascent axoneme within the cytoplasm, covered by a double lipid bilayer. The ciliary vesicle eventually fuses with the plasma membrane in a process mediated by the formation of membrane deforming protein EHD1- and PACSIN1-decorated tubular membranes connecting these membrane domains [25]. This releases the nascent cilium which is now covered by a single membrane, the ciliary membrane [23]. All proteins required for cilia assembly and function must be trafficked into and out of the cilium from the cytoplasm, a process achieved by the intraflagellar transport (IFT) proteins and kinesin/dynein microtubule motors [26].

The process of cilia disassembly is less well characterised but is known to rely on microtubule destabilisation (including by deacetylation) and disassembly for which Aurora kinase A and its substrate histone deacetylate 6 are key players (Figure 3(iv,v)) [27,28]. Actin-dependent decapitation of the cilia tip, whereby the tip is released from the cell as a vesicle, has also recently emerged as a cilia disassembly-promoting event by removing IFT components [29], and is described in further detail below in the context of its regulation by phosphoinositides.

### Ciliopathy syndromes

Mutations in ciliary proteins cause a phenotypic spectrum of developmental disorders known as ciliopathy syndromes, including Joubert syndrome, Meckel–Gruber syndrome, nephronophthisis and Bardet–Biedl syndrome (BBS), among others [30]. The importance of primary cilia in development and homeostasis is also illustrated by mouse models with deletion of key cilia genes which are associated with severe phenotypes and often embryonic lethality [30,31]. Characteristic ciliopathy disease and mouse model phenotypes include cognitive impairment, polycystic kidneys, polydactyly, blindness, obesity, infertility and frequently result from dysregulation of cilia signalling-dependent developmental patterning [17,30].

### Primary cilia signalling

One of the most widely studied functions of primary cilia is as a key compartment for multiple signalling pathways such as Hedgehog, planar cell polarity, WNT, G protein-coupled receptors (GPCRs) and receptor tyrosine kinases (RTKs) such as Platelet-Derived Growth Factor-Receptor (PDGFR), among others [32–36].

**Figure 4.**
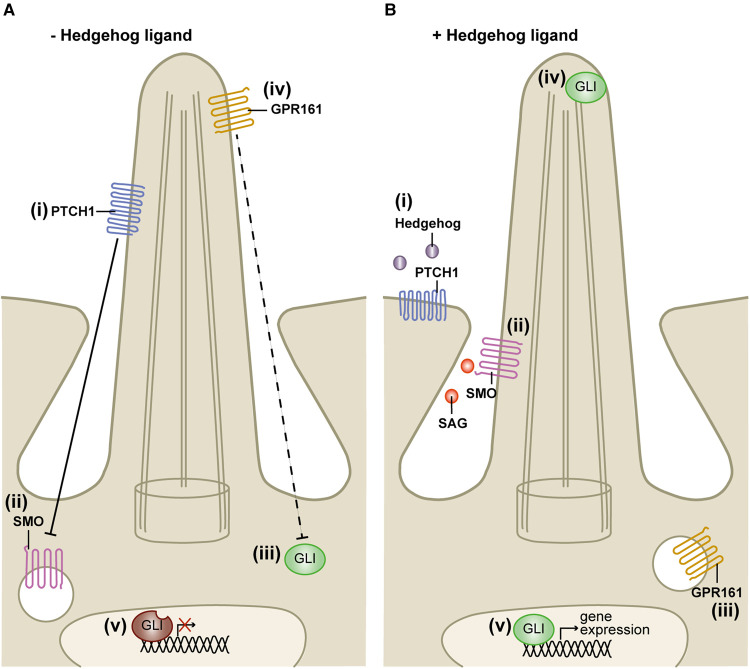
Hedgehog signalling is initiated at primary cilia. (**A**) In the absence of Hedgehog ligand, (**i**) its receptor PTCH localises to the primary cilium and represses the pathway by (**ii**) sequestering the GPCR-like protein SMO out of the cilium. (**iii**) The GLI transcription factors are sequestered in the cytoplasm. (**iv**) The GPCR GPR161 localises to cilia and supports the processing of GLI to the proteolytically processed transcriptional repressor. (**v**) GLI repressors translocate to the nucleus to inhibit target gene expression. (**B**) (**i**) Hedgehog ligand binding to PTCH1 results in PTCH1 movement out of the cilium and (**ii**) de-repression of SMO, which now accumulates in the ciliary membrane. SMO may also be directly activated by the small molecule ligand SAG. (**iii**) These events result in exit of GPR161 from the cilia and (**iv**) GLI accumulation at the cilia tip where it becomes activated. (**v**) Active GLI then translocates to the nucleus to promote Hedgehog target gene expression.

Importantly, many models with cilia dysfunction exhibit defective Hedgehog-dependent patterning (reviewed in [16]). Canonical Hedgehog signalling is absolutely dependent on a functional primary cilium, with its complex regulation shown in Figure 4 [32]. Hedgehog signalling ultimately results in a gene expression program mediated by the GLI transcriptional factors (GLI1, GLI2 and GLI3). In unstimulated cells GLI3 is proteolytically-processed to a transcriptional repressor (Figure 4A(v)) [37]. Stimulation of the pathway results in activation of the full-length transcriptional activator GLI2 (Figure 4B(v)) [38]. GLI1 is also a transcriptional activator but is only expressed following GLI2 activation forming a positive feedback loop [39].

In the absence of Hedgehog ligand, the inhibitory receptor Patched 1(PTCH1) localises to the cilium and represses the GPCR-like protein Smoothened (SMO) via an incompletely understood mechanism possibly requiring ion gradients and cholesterol metabolism (Figure 4A(i,ii)) [40,41]. A negative regulator, called Suppressor of fused (SUFU), binds the GLI transcription factors and sequesters the full length activator forms in the cytosol (Figure 4A(iii)) [42]. The GPCR GPR161 is another negative regulator which localises to the cilium to promote GLI3 repressor formation by signalling through Gα_s_/cAMP/PKA (Figure 4A(iv)) [36]. The GLI3 repressor localises to the nucleus to inhibit target gene expression (Figure 4A(v)) [37].

Binding of a Hedgehog ligand (Sonic, Indian or Desert Hedgehog) to PTCH1 results in PTCH1 movement out of the cilium (Figure 4B(i)) and de-repression of SMO which is activated and accumulates at the ciliary membrane (Figure 4B(ii)) [40,43,44]. Cilia localised active SMO removes GPR161 from cilia (Figure 4B(iii)) [36] and promotes the processing of GLI2 to a transcriptional activator at the cilia tip (Figure 4B(iv)), which traffics to the nucleus, overriding the transcriptional repressor activity of GLI3 [38]. Active GLI2 promotes target gene expression (Figure 4B(v)), including expression of *GLI1* and *PTCH1,* forming positive and negative feedback loops, respectively, as well as many cell cycle and cell type-specific patterning regulators [38,45,46].

Importantly, the cilium not only acts as a hub to localise and enable Hedgehog signal transduction, in the absence of ligand it also restrains the pathway via processing of GLI3 to a transcriptional repressor [47–49]. Therefore loss of cilia function phenotypes are often more complex than simply Hedgehog repression and may include the typical Hedgehog gain-of-function polydactyly phenotype [16,32,50].

## Phosphoinositides and their binding domains

PtdIns consists of a fatty acid and glycerol backbone with a D-myo-inositol-1-phosphate headgroup which is modified at the D3, D4 or D5 positions by lipid kinases and phosphatases to generate up to seven distinct lipid molecules (Figure 1A).

Non-phosphorylated PtdIns, the most abundant species, constitutes 15% of total cellular phospholipids and is synthesised in the endoplasmic reticulum prior to trafficking to various organelles for phosphorylation by resident kinases [51]. Even the most abundant phosphorylated phosphoinositide species are present at 10–20 fold lower levels compared with PtdIns [51]. The local phosphoinositide composition of an organelle membrane or subdomain is tightly regulated by phosphorylation/dephosphorylation events and lipid transport proteins. Specific phosphoinositides localise to the cytoplasmic surface of the plasma membrane and membranous organelles thereby defining membrane identity (Figure 1B).

PtdIns(4,5)P_2_ is the most abundant species at the plasma membrane where it co-ordinates endocytosis and actin dynamics and serves as a substrate for phospholipase C and class I phosphoinositide 3-kinases (PI3Ks) (Figure 1B) [52–57]. PtdIns(3)P and PtdIns(4)P define endosomes and the trans-Golgi network, respectively (Figure 1B) [58,59]. In contrast with these constitutively-present membrane lipids, PIP_3_ and PtdIns(3,4)P_2_ are produced only transiently following growth factor stimulation (Figure 1B) [13,55].

Phosphoinositides dictate membrane identity and act by recruiting effector proteins via their lipid binding domains such as the Pleckstrin Homology (PH), FYVE, Phox and C2 domains. Below, we briefly discuss PH, C2 and the related but little characterised B9 domains, which are found in cilia-localised proteins.

PH domains are a widely distributed protein fold present in many diverse proteins [60]. A small subset of PH domains binds lipids, including PIP_3_, PtdIns(3,4)P_2_, PtdIns(4)P and/or PtdIns(4,5)P_2_, [61,62]. One of the most well-studied phosphoinositide-binding PH domains is that in the AKT serine/threonine kinase, which facilitates the recruitment of this kinase to the plasma-membrane following the production of PIP_3_/PtdIns(3,4)P_2_ by class I PI3Ks. Membrane recruitment brings AKT in proximity to the already membrane-bound PDK1 resulting in AKT phosphorylation on threonine 308 by PDK1 [63–65]. Both phosphorylated PDK1 and AKT have been identified at the cilia base [7,66].

C2 domains are found in at least 200 proteins, many of which regulate signalling and vesicle trafficking [67]. The C2 domain is an ∼130 amino acid protein fold, some of which mediate lipid binding, although with less specificity compared with most PH domains [67,68]. Many cilia transition zone proteins contain C2 domains which are hypothesised to mediate protein-membrane interactions [20]. However, for many C2 domains the lipid binding properties and whether they indeed bind phosphoinositides at all remains unclear. For example, it is suggested that the negative charge of the C2 domains of the cilia-associated RPGRIP1L protein render it unlikely to bind phosphoinositides, but this has not been directly tested [69].

B9 domains are closely related to C2 domains and are found specifically in the transition zone proteins B9D1, B9D2 and MKS1, but have not yet been characterised in detail and their phosphoinositide binding properties are unknown [20,70].

## Cilia-associated phosphoinositide kinases and phosphatases

**Figure 5.**
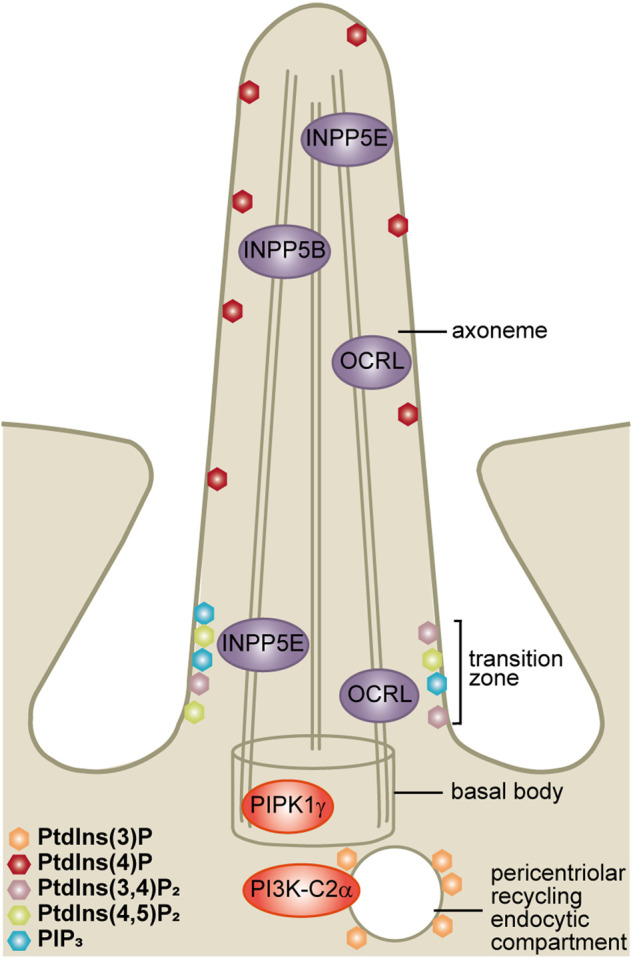
Phosphoinositide map at primary cilia. The phosphoinositides localise to discrete subcompartments of the ciliary membrane. PtdIns(4)P is the major phosphoinositide in the ciliary membrane. The transition zone membrane at the cilia base is a ‘hot-spot’ enriched for multiple phosphoinositides including PtdIns(4,5)P_2_, PIP_3_ and PtdIns(3,4)P_2_. PtdIns(3)P is associated with the pericentriolar recycling endocytic compartment. The phosphoinositide regulatory enzymes PI3K-C2α, PIPKIγ, INPP5E, OCRL and INPP5B also localise to specific ciliary subdomains in proximity to their substrates and products.

The following sections will introduce the cilia-associated phosphoinositide lipid kinases and phosphatases, summarising the evidence that they regulate cilia biology under normal conditions and are the subject of disease-associated mutations in humans (Figure 5 and Table 1).

**Table 1:** Cilia associated PI regulatory enzymes, their localisation at cilia and cilia associated substrates and products

Class	Enzyme	Substrate	Product	Cilia Localisation	References
Kinases	PI3K-C2α	PtdIns	PtdIns(3)P	Pericentriolar recycling endocytic compartment	[9]
	PIPKIγ	PtdIns(4)P	PtdIns(4,5)P_2_	Centrosome	[10]
Phosphatases	INPP5E	PtdIns(4,5)P_2_ PIP_3_	PtdIns(4)P PtdIns(3,4)P_2_	Cilia axoneme Transition zone (in some cells)	[6,11,12]
	OCRL	PtdIns(4,5)P_2_ PIP_3_	PtdIns(4)P PtdIns(3,4)P_2_	Cilia axoneme Cilium base	[77,107]
	INPP5B	PtdIns(4,5)P_2_ PIP_3_	PtdIns(4)P PtdIns(3,4)P_2_	Cilia axoneme	[119]

### Regulation of ciliary phosphoinositides by PI-kinases

#### PI3K-C2α

PI3K-C2α is a ubiquitously expressed class II PI3K which regulates endocytic trafficking [71]. PI3K-C2α phosphorylates PtdIns and PtdIns(4)P to produce PtdIns(3)P and PtdIns(3,4)P_2_ [9,72], with its substrate specificity possibly depending on the relative abundance of PtdIns and PtdIns(4)P at specific membrane sites. PI3K-C2α localises to the cilia-associated pericentriolar recycling endocytic compartment (Figure 5) and is proposed to regulate cilia by modulating local PtdIns(3)P levels, as described in further detail below [9]. Deletion or knockdown of *Pik3c2a* (which encodes PI3K-C2α) reduces cilia length and impairs the trafficking of transmembrane proteins into cilia [9,73]. Homozygous *Pik3c2a* deletion in mice is embryonically lethal at embryonic day (E)10.5–E11.5 with a phenotype characteristic of primary cilia and Hedgehog pathway dysfunction including reduced embryo size, failed embryonic turning and cardiac tube looping [9,74]. These embryos also show a phenotype of defective angiogenesis which is likely unrelated to the primary cilia defect [9,74]. Hedgehog ligand-stimulated Hedgehog signalling is impaired in *Pik3c2a*-depleted embryos and mouse embryonic fibroblasts (MEFs) [9]. Furthermore, heterozygous *Pik3c2a* deletion in several cystic kidney disease models exacerbates renal cyst formation, a typical ciliopathy phenotype [73].

A novel human syndrome defined by skeletal malformations, short stature, cataracts and developmental delay was recently associated with homozygous *PIK3C2A* mutations [75]. All mutations identified to date are thought to be loss-of-function and result in a loss of protein expression [75]. Many features of this syndrome are similar to classical ciliopathy syndromes [30] and closely resemble Lowe's syndrome, a ciliopathy-associated disorder with mutations in the cilia-localised 5-phosphatase *OCRL* discussed below [76,77]. Similar to *Pik3c2a*-null cells, *PIK3C2A* mutant human fibroblasts exhibit reduced cilia length, suggesting that this *PIK3C2A* mutant syndrome may represent a new ciliopathy [75].

#### PIPKIγ

Type Iγ PtdIns(4)P 5-kinase (PIPKIγ) localises to the mother centriole (cilia basal body) in ciliated (Figure 5) and non-ciliated cells and phosphorylates PtdIns(4)P to produce PtdIns(4,5)P_2_ [10,78]. This kinase has also been shown to localise to and regulate endosomes and cell adhesion junctions and is required for signalling events [79–85]. Six PIPKIγ splice isoforms have been detected with distinct subcellular localisations and functions [86–90]. In some contexts, such as at the non-ciliated centriole, PIPKIγ isoform i3 operates via a kinase-independent, scaffold-dependent mechanism [78]. In several cell lines PIPKIγ depletion arrests ciliogenesis and reduces cilia length in cells that do form cilia, in a kinase-dependent manner, although this study did not examine whether a single or multiple isoforms contribute to this function [10]. Furthermore, deletion of the sole Type Iγ PtdIns(4)P 5-kinase homologue in *Caenorhabditis elegans* results in defective cilia function [10]. Three mouse models that are null for *Pip5k1c* (which encodes PIPKIγ) have been reported [91–93]. Mice homozygous for a gene trap allele in which the first 32 PIPKIγ amino acids are fused to β-Gal are embryonically lethal with defective neural tube closure, a ciliopathy phenotype, although ciliogenesis and cilia function have not be examined in this model [91]. In contrast, the other murine *Pip5k1c* knockout (KO) models survive to birth and die as neonates, with no evidence of a ciliopathy phenotype [92,93]. The reasons for the distinct phenotypes in these models remain unknown [91–93]

### Regulation of ciliary phosphoinositides by inositol PI 5-phosphatases

In mammals, the inositol polyphosphate 5-phosphatases are a family of ten members that play non-redundant roles in cell biology, with three members, INPP5E, OCRL and INPP5B having established functions at cilia by acting as major regulators of their substrates PtdIns(4,5)P_2_ and PIP_3_ at this site [4–6,94,95]. This review will focus on these cilia-associated phosphatases. An additional 5-phosphatase, SHIP2, has also been shown to regulate cilia formation and length but not via direct regulation of ciliary phosphoinositides [96] and will therefore not be discussed here.

#### INPP5E

INPP5E hydrolyses the 5-phosphate from PtdIns(4,5)P_2_ and PIP_3_ with the greatest activity towards the latter lipid *in vitro* [97,98] and localises to the cilia axoneme, transition zone (Figure 5) and the Golgi [6,11,12,98].

INPP5E mutations cause two human ciliopathy syndromes, namely Joubert syndrome (OMIM #213300) and MORM (mental retardation, obesity, congenital retinal dystrophy and micropenis in males; OMIM #610156) [11,12].

Joubert syndrome is an autosomal recessive disorder defined by a mid-brain malformation (the ‘molar tooth sign’) and associated with mental retardation, retinal degeneration, apnea and cystic kidneys [12,99,100]. Loss-of-function mutations in more than 30 genes have been identified in Joubert syndrome to date. *INPP5E* Joubert syndrome mutations cluster in or around the 5-phosphatase domain and the mutant proteins assayed show reduced *in vitro* 5-phosphatase activity towards both PtdIns(4,5)P_2_ and PIP_3_ [12,101].

*INPP5E* is the only gene known to be mutated in MORM, a rare congenital disorder that resembles other ciliopathy syndromes and has been identified in a consanguineous family [11]. The MORM mutation results in deletion of the 18 C-terminal amino acids of INPP5E including the CAAX motif, which is important for ciliary membrane localisation, but does not affect 5-phosphatase activity [11].

In experimental model systems such as zebrafish, knockdown of *inpp5e* induces pronephric cysts, microphthalmia, pericardial edema and kinked tail phenotypes, characteristic of cilia dysfunction and associated with reduced ciliated cells and cilia length in the Kupffer's vesicle and pronephric ducts [102]. *Inpp5e* KO mice are embryonically lethal with a classical ciliopathy phenotype including exencephaly, anophthalmia, polydactyly, polycystic kidneys, pulmonary hypoplasia, cleft palate, oedema and ossification delays [6,11]. Renal-specific *Inpp5e* deletion results in severe polycystic kidneys and renal failure with morbidity by postnatal day 21 [103,104]. *Inpp5e*-null cells exhibit reduced cilia stability [11] and consistent with multiple aspects of the phenotype, a diminished *in vitro* and *in vivo* Hedgehog pathway response [4–7]. Significantly, expression of a constitutively active Smoothened mutant in the context of *Inpp5e* deletion during embryonic development dramatically rescues anophthalmia and exencephaly and partially rescues the oedema, cleft palate and polydactyly, indicating that these *Inpp5e*-null phenotypes are caused by repressed Hedgehog signalling [6].

#### OCRL

OCRL hydrolyses PtdIns(4,5)P_2_, PIP_3_ and PtdIns(3,5)P_2_, with the greatest activity towards PtdIns(4,5)P_2_ [105,106]. OCRL localises to the base of the cilium, the axoneme (Figure 5) and the endocytic network (the Golgi, early endosomes, clathrin coated vesicles and autolysosomes) [77,107–111]. While OCRL has a well-established role in endocytic trafficking and actin cytoskeletal regulation (reviewed in [111]), its role at cilia and in regulation of ciliary phosphoinositides is just beginning to emerge [77,95,107,112]. OCRL is mutated in oculocerebrorenal syndrome of Lowe (Lowe syndrome) (OMIM #309000) [76] and Dent 2 disease (OMIM #300555) [113]. All disease-associated mutations are loss-of-function and reduce its PtdIns(4,5)P_2_ 5-phosphatase activity, with the level of residual protein and severity of the cell biological defects appearing to differentiate the more severe Lowe syndrome from the relatively mild, largely renal-specific, Dent 2 disease [113–115].

Lowe syndrome subjects exhibit Fanconi syndrome (low-molecular-weight proteinuria, aminoaciduria and hypercalciuria), mental retardation, maladaptive behaviour and congenital cataracts. Lowe syndrome does not completely phenocopy a true ciliopathy syndrome but exhibits some key similarities and is therefore suggested to be a ciliopathy-associated disease [112,116]. Evidence supporting Lowe syndrome as a ciliopathy includes the involvement of the same major organs (brain, kidney and eye), overlapping phenotypes (cataracts, hypotonia and polyuria) and the presence of defects in length, assembly/disassembly and function of cilia in mutant and KO/knockdown cells [77,107,112,115]. Furthermore, *ocrl* knockdown zebrafish are lethal with ciliopathy phenotypes including defective body-axis asymmetry and curvature, pericardial oedema, hydrocephalus, microphthalmia, microlentis and pronephric cysts [77,107,112]. Interestingly, *Ocrl* deletion in mice has no phenotypic effects due to compensation by the *Ocrl* homologue *Inpp5b*, with *Ocrl;Inpp5b* double KO mice exhibiting embryonic lethality [117]. Reconstitution of *Ocrl;Inpp5b* double KO mice with human *INPP5B* rescued lethality but still resulted in a Lowe syndrome-like phenotype including aminoaciduria, proteinuria and growth retardation, indicating lack of full functional conservation between mouse and human *INPP5B* [118].

#### INPP5B

Similar to its closely related homologue OCRL, INPP5B also contributes to regulation of ciliary phosphoinositides [117,119]. INPP5B localises to the cis-Golgi, the endoplasmic reticulum-to-Golgi intermediate compartment, endosomes, phagosomes and the primary cilium (Figure 5) and hydrolyses PtdIns(4,5)P_2_ and PIP_3_ [119–124]. Depletion of *INPP5B* reduces ciliated cell number and cilia length [119]. *inpp5b* knockdown zebrafish also exhibit a ciliopathy phenotype consisting of microphthalmia, kinked tail and body axis asymmetry defects [119]. The molecular mechanisms by which INPP5B regulates cilia biology have been little characterised to date.

## Ciliary phosphoinositide map

The ciliary membrane is continuous with the plasma membrane, however, it is becoming increasingly clear that it exhibits a distinct phosphoinositide composition [4–8]. How the different ciliary lipid membrane domains are established and maintained remains incompletely understood but this may relate to the specific localisation of ciliary lipid kinases and/or phosphatases such as PI3K-C2α [9], PIPKIγ [10], INPP5E [11], OCRL [77,107] and INPP5B [119] (Figure 5 and Table 1), or the presence of a physical diffusion barrier [125,126]. In support of a membrane diffusion barrier, glycosylphosphatidylinositol-tagged yellow fluorescent reporter proteins targeted to the apical membrane of epithelial cells are excluded from cilia [125]. Furthermore, in *Caenorhabditis elegans* mutation of the transition zone component *mks5* resulting in defective barrier function allows PtdIns(4,5)P_2_ to abnormally accumulate along the length of the cilium [126].

PtdIns(4,5)P_2_, PtdIns(4)P, PIP_3_ and PtdIns(3,4)P_2_ localise to specific sub-regions of the cilia membrane, with PtdIns(3)P found at the cilia-associated pericentriolar recycling endocytic compartment (Figure 5 and Table 2) [4–9,95,126].

**Table 2:** Localisation and function of phosphoinositides at primary cilia

Cilia compartment	Phosphoinositides	Proposed function	References
Centriole (in non-ciliated cells)	PtdIns(4)P	inhibition of ciliogenesis	[10]
Pericentriolar recycling endocytic compartment	PtdIns(3)P	Rab8 activation, ciliogenesis, cilia trafficking	[9]
Ciliary membrane	PtdIns(4)P	Unknown	[4,5]
	PtdIns(4,5)P_2_	recruitment of TULP3 and GPCR trafficking	[4,5]
		actin polymerisation and cilia decapitation	[29]
Transition zone	PIP_3_	transition zone integrity and barrier function	[6]
		cilia stability/disassembly	[7]
	PtdIns(4,5)P_2_	transition zone integrity and barrier function	[6]
	PtdIns(3,4)P_2_	Unknown	[7]

PtdIns(4,5)P_2_ is the most extensively studied ciliary phosphoinositide in a range of species and cell types [4–8,95,126]. The majority of studies conclude that PtdIns(4,5)P_2_ is concentrated at the ciliary base, however, subtle differences have been described [4–8,95,126]. Indeed, some reports contend that PtdIns(4,5)P_2_ localises to the proximal end of the cilium [5] or the base [8,126], whereas other studies show it colocalises with transition zone markers [6,7,95] and one study suggested enrichment at the cilia tip but only following deletion of INPP5E [4]. These differences may relate to species or cell types used, but are most likely due to technical differences in the phosphoinositide detection methods used (Box 1). Although, a common interpretation from these studies is that PtdIns(4,5)P_2_ is depleted at the ciliary membrane relative to the plasma membrane, consistent with the essential role multiple 5-phosphatases play in cilia biology [4–8,95,126].

The localisation of the other phosphoinositides is less contentious, possibly due to the fact that they have been examined in less studies to date. PIP_3_ and PtdIns(3,4)P_2_ localise to the transition zone [6,7], with PtdIns(4)P being continuous along the cilia membrane [4,5,95] and PtdIns(3)P at the pericentriolar recycling endocytic compartment at the cilia base (Figure 5 and Table 2) [9]. Given the differences in PtdIns(4,5)P_2_ localisation between studies and that relatively fewer groups have assessed the other ciliary phosphoinositides it is likely that all species will benefit from a more comprehensive multi-approach analysis to confirm their localisations.

## Phosphoinositides and their functions in ciliary subcompartments

Below, we will explore each of the ciliary subcompartments (centrosome, pericentriolar recycling endocytic compartment, ciliary membrane and transition zone), describing which phosphoinositides localise to these sites, examine their functions in cilia biology and highlight outstanding questions. Table 2 provides a summary of this section.

### The centrosome: in non-ciliated cells centrosomal PtdIns(4)P inhibits ciliogenesis and is removed to licence transition zone formation and axoneme elongation

During ciliogenesis the mother centriole docks with the plasma membrane [127] (Figure 3(iii)) where the microtubule capping protein CP110 must be removed from the distal end of the centriole to enable transition zone formation and axoneme elongation [128,129]. CP110 is removed from the mother centriole by tau-tubulin kinase-2 (TTBK2), which itself is recruited to the centriole by the distal appendage protein CEP164 [130,131]. Both TTBK2 and CEP164 contain phosphoinositide binding sites that interact with PtdIns(4)P, with lipid binding disrupting the TTBK2/CEP164 complex [10].

Notably, in non-ciliated cells PtdIns(4)P localises to the centrosome and is removed by phosphorylation upon serum starvation and the induction of ciliogenesis [10]. Therefore centrosomal PtdIns(4)P is proposed to inhibit local CEP164-mediated TTBK2 recruitment and thereby suppress cilia formation in non-ciliated conditions [10]. Upon serum starvation, PtdIns(4)P is phosphorylated at the centrosome to PtdIns(4,5)P_2_, enabling CEP164 to recruit TTBK2, promoting removal of CEP110 from the distal centriole and licencing the cell competent for ciliogenesis [10].

Centrosomal PtdIns(4)P levels are tightly regulated by the opposing actions of PIPKIγ and INPP5E [10]. PIPKIγ localises to the mother centriole in ciliated cells where it is proposed to deplete the levels of PtdIns(4)P by phosphorylating it to PtdIns(4,5)P_2_, whereas INPP5E is observed at the centriole only in non-ciliated cells to produce PtdIns(4)P and thereby inhibit ciliogenesis [10]. INPP5E then moves into the axoneme once the cell assembles the cilium, thereby depleting PtdIns(4)P levels at the centrosome [10,12]. Indeed, PIPKIγ depletion and INPP5E overexpression, both of which are expected to increase local PtdIns(4)P, suppress ciliogenesis and in PIPKIγ knockdown cells ciliogenesis was halted between the centriole docking and transition zone formation steps [10].

#### Unanswered questions

The antibodies and siRNAs used in the studies above recognise all PIPKIγ splice isoforms and the authors do not state which isoform was used to generate the overexpression plasmids, therefore it is unknown whether one or more isoforms contribute to ciliogenesis [10]. Similarities between the phenotype of one of the *Pip5k1c* (encoding PIPKIγ) KO models and ciliopathies supports the contention that PIPKIγ is required for ciliogenesis, whereas the two other *Pip5k1c* KO models reported do not show ciliopathy phenotypes, raising questions on how to interpret these mouse phenotypes [91–93].

This lipid interconversion model also appears inconsistent with the phenotype of *Inpp5e*-null mice and the numerous studies showing that INPP5E is required for cilia maintenance [4–7,11,12,29,102,132]. Perhaps, INPP5E negatively regulates the initial stages of ciliogenesis but then stabilises pre-established cilia. The fact that the *Inpp5e*-null phenotype is characteristic of a cilia loss-of-function model may suggest INPP5E's predominant role is to stabilise cilia/enable normal cilia function and the loss of ciliated cells following ectopic INPP5E expression *in vitro* may be an overexpression artefact.

An intriguing question is the precise localisation of this PtdIns(4)P pool. The centrosome is a non-membranous protein structure. It could be hypothesised that the PtdIns(4)P may bind to lipid transfer proteins at this site or that PtdIns(4)P-decorated vesicles associate with the centrosome.

### Pericentriolar recycling endocytic compartment: local PtdIns(3)P controls cilia length and subcellular trafficking of cilia proteins

A specific pool of PtdIns(3)P is produced by PI3K-C2α at the pericentriolar recycling endocytic compartment, a Rab11-positive endosomal structure at the base of the cilium (Figure 5) [9]. Multiple Rab GTPases including Rab11, Rab8 and its guanine nucleotide exchange factor (GEF) Rabin8 are essential for protein entry into the cilium and cilia lengthening. Rab11 promotes Rabin8 GEF activity and thereby stimulates Rab8 activation [133–136]. PtdIns(3)P recruits and activates Rab11 at the pericentriolar recycling endocytic compartment and promotes Rab8 accumulation at cilia [9]. Depletion of this PtdIns(3)P pool by *Pik3c2a* deletion or knockdown reduces cilia length and impairs the trafficking of transmembrane proteins SMO and polycystin-2 into cilia [9,73]. Importantly, PI3K-C2α generates both PtdIns(3)P and PtdIns(3,4)P_2_ but only the production of PtdIns(3)P is required for Rab11 recruitment and thereby regulation of this cilia trafficking pathway [9].

The importance of the cilia-associated PtdIns(3)P pool is highlighted by the ciliopathy phenotype and repressed cilia signalling of ubiquitous and renal-specific *Pik3c2a* KO mouse models [9,73]. These phenotypes are consistent with the impaired Smoothened and polycystin-2 cilia trafficking [9,73]. Furthermore, fibroblasts from subjects with the novel *PIK3C2A* mutant ciliopathy-like syndrome exhibit shorter cilia with reduced PtdIns(3)P and Rab11 levels at the cilia base [75]. In renal epithelial cells this cilia base endosomal PtdIns(3)P pool is increased by shear stress in a PI3K-C2α-dependent manner to recruit autophagy mediators and thereby activates a non-canonical shear stress-induced autophagy program [137].

#### Unanswered questions

There are many outstanding questions regarding the pericentriolar recycling endocytic compartment. This includes whether (1) this indeed constitutes a separate subcellular compartment, distinct from the conventional recycling endosome; (2) why incoming cargo appears to not directly traffic from the Golgi or recycling endosomes into the cilium and (3) whether a specific type of carrier needs to be formed in order to allow entry into the cilium.

### The ciliary membrane: a local PtdIns(4)P/PtdIns(4,5)P_2_ balance controls receptor recruitment to cilia

PtdIns(4)P localises along the ciliary membrane, with little or no PtdIns(4,5)P_2_ localising to this site (Figure 5) [4,5,95]. However, loss of *INPP5E* in neural stem cells and MEFs depletes the ciliary membrane PtdIns(4)P pool and induces a dramatic accumulation of PtdIns(4,5)P_2_, suggesting the major pathway for PtdIns(4)P production is via dephosphorylation of PtdIns(4,5)P_2_ at the 5-position [4,5,95]. Indeed, PIPKIγ is enriched at the base of the cilium where it could produce PtdIns(4,5)P_2_ [4]. A similar phenotype of PtdIns(4)P loss and PtdIns(4,5)P_2_ build-up at the cilium is observed in fibroblasts from human Lowe syndrome subjects and in MEFs from the human *INPP5B* reconstituted *Ocrl;Inpp5b* co-deletion Lowe syndrome mouse model [95].

In contrast with the centrosomal PtdIns(4)P pool, the role of PtdIns(4)P at the ciliary membrane is yet to be uncovered, although it is possible that this lipid may not have a specific function in this compartment and may simply be the degradation product remaining from PtdIns(4,5)P_2_ hydrolysis. However, the aberrant accumulation of PtdIns(4,5)P_2_ at the cilia membrane has a major impact on cilia signalling and stability and is proposed to contribute to the ciliopathy phenotypes observed with *INPP5E* mutation or deletion [4,5,29].

**Figure 6.**
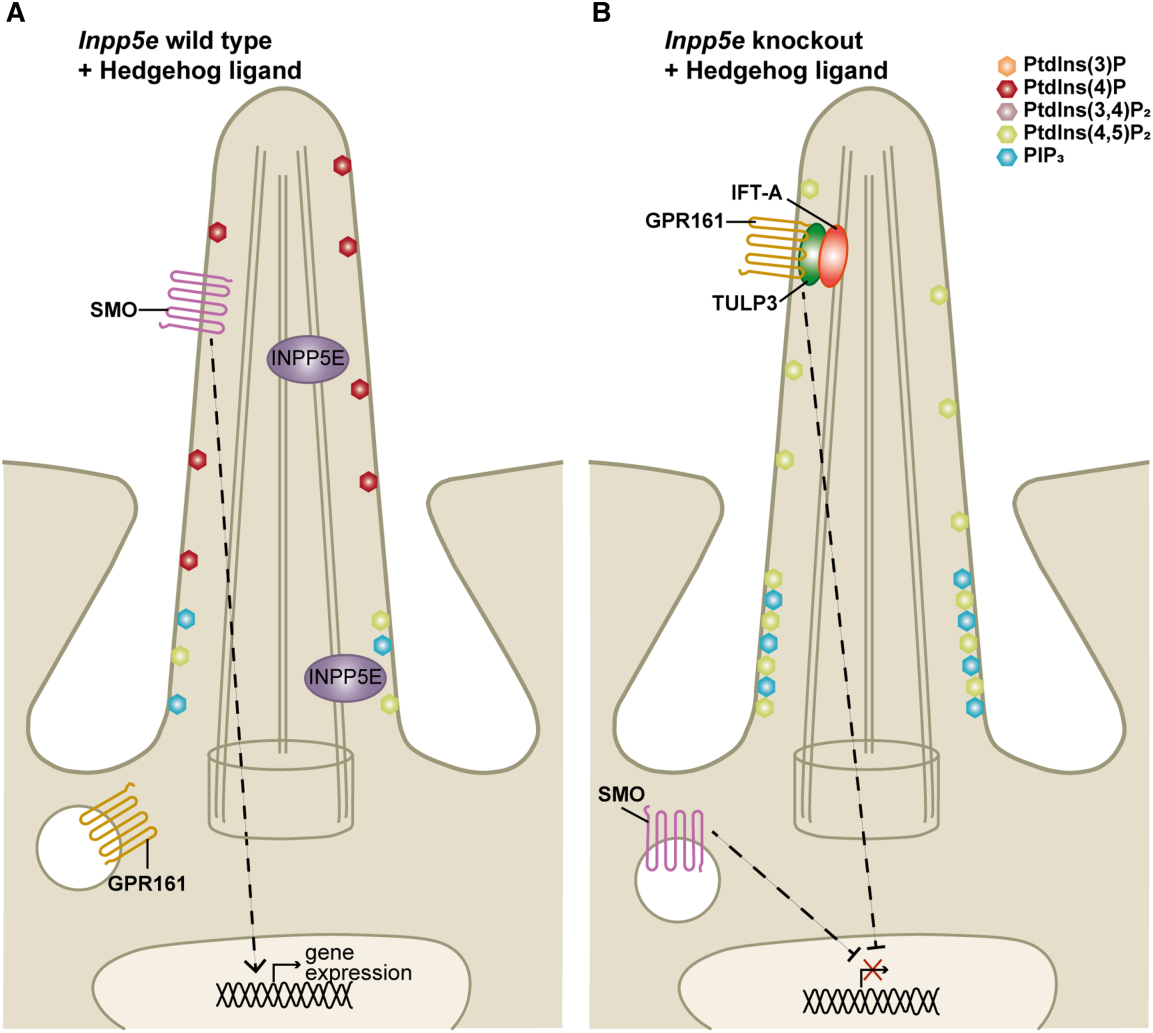
INPP5E regulates Hedgehog signalling via ciliary phosphoinositide metabolism. (**A**) INPP5E localises to the cilia axoneme and transition zone. In the axoneme INPP5E prevents the accumulation of PtdIns(4,5)P_2_ via hydrolysis to PtdIns(4)P. At the transition zone INPP5E regulates the local PtdIns(4,5)P_2_ and PtdIns(3,4,5)P_3_ levels. Upon Hedgehog stimulation of wild type cells SMO accumulates at the cilium promoting ciliary exit of the negative regulator GPR161 and subsequently Hedgehog target gene expression. (**B**) In the axoneme of *Inpp5e* knockout cilia PtdIns(4,5)P_2_ accumulates. TULP3 binds the increased ciliary PtdIns(4,5)P_2_ inducing GPR161 accumulation even in the presence of Hedgehog ligand stimulation. PtdIns(4,5)P_2_ and PIP_3_ accumulate in the *Inpp5e*-null transition zone upon Hedgehog pathway activation which is associated with disruption of the transition zone complexes and compromised diffusion barrier retention function. Therefore SMO fails to concentrate in *Inpp5e*-null cilia. These events contribute to a reduced transcriptional response to Hedgehog stimulation.

In both neural stem cells and MEFs, *Inpp5e* deletion leads to PtdIns(4,5)P_2_ accumulation at the ciliary membrane [4,5]. The phosphoinositide binding protein Tubby-like protein 3 (TULP3) acts as a molecular bridge linking ciliary membrane proteins to the IFT machinery, thereby enabling protein localisation to primary cilia, a function dependent on the phosphoinositide binding domain of TULP3 [138,139]. TULP3 binds to PtdIns(4,5)P_2_, PIP_3_ and PtdIns(3,4)P_2_ via its Tubby domain [139,140]. Ciliary TULP3 levels are increased in *Inpp5e* KO cells suggesting increased ciliary PtdIns(4,5)P_2_ induces TULP3 accumulation [4,5]. Significantly, GPR161 is a TULP3 cargo and an important negative regulator of Hedgehog signalling (Figure 4A(iii)) [36]. Like TULP3, GPR161 aberrantly accumulates in *Inpp5e*-null cilia in the absence and presence of Hedgehog pathway stimulation, which directs internalisation in wild-type cells [4,5,36]. Therefore accumulation of PtdIns(4,5)P_2_ in cilia following *Inpp5e* deletion is proposed to recruit elevated levels of the TULP3/GPR161 complex to cilia and thereby repress Hedgehog signalling (Figure 6) [4,5]. Regulation of Hedgehog signalling via this model could contribute to the Hedgehog-dependent phenotypes observed in *Inpp5e*-null embryos [6,11].

Primary cilia disassembly is induced in cell culture by serum or growth factor stimulation as cells re-enter the cell cycle [21]. Following serum stimulation, INPP5E exits the cilium creating a situation similar to *Inpp5e*-null cells which are hypersensitive to disassembly stimuli [11,29]. As serum stimulation removes INPP5E from cilia, PtdIns(4,5)P_2_ is no longer hydrolysed and accumulates [29]. Outside the cilium PtdIns(4,5)P_2_ is well-established to promote actin polymerisation (reviewed in [141]). Cilia are normally devoid of actin, however, following serum stimulation actin polymerises at the site of maximal PtdIns(4,5)P_2_ signals and mediates an event termed cilia decapitation, whereby the cilia tip buds off and is released from the cell (Figure 3(iv)) [29]. Ciliary components are released in these vesicles and the process promotes cilia disassembly and cell cycle re-entry [29].

#### Unanswered questions

TULP3 not only binds PtdIns(4,5)P_2_, but has also been reported to interact with PIP_3_ and PtdIns(3,4)P_2_ [139]. Given that both of these lipids localise to the transition zone, the potential role of PIP_3_ and PtdIns(3,4)P_2_ in TULP3 and its target recruitment to cilia is an interesting question to consider [6,7]. Furthermore, cilia decapitation, sometimes also referred to as ectocytosis, removes GPCRs from the cilium thereby regulating cilia signalling [142]. Similar to decapitation-induced cell cycle re-entry, this process is actin-dependent suggesting a similar molecular mechanism [29,142]. However, a role for phosphoinositides such as PtdIns(4,5)P_2_ in this process has not been examined.

### The transition zone

The transition zone membrane is a phosphoinositide enrichment ‘hot-spot’ (Figure 5). At this site phosphoinositides are principally regulated by phosphorylation/dephosphorylation of the phosphate at the 5-position of the inositol ring. In the following sections we describe the important functions of the transition zone-associated phosphoinositides [PtdIns(4,5)P_2_, PIP_3_ and PtdIns(3,4)P_2_] and their regulation. The transition zone is the only cilia subdomain so far to be associated with PIP_3_ and PtdIns(3,4)P_2_, the products of class I PI3K signalling [6,7].

#### Transition zone PtdIns(4,5)P_2_ and PIP_3_ respond to Hedgehog signalling and regulate diffusion barrier function

Discrete pools of PtdIns(4,5)P_2_ and PIP_3_ localise to the cilia transition zone in MEFs, granule cell progenitor (GCP)-like tumour cells and hTERT-retinal pigment epithelial 1 cells where they co-localise with the transition zone component TCTN1 and regulate transition zone function (Figure 5) [6,7]. Transition zone PtdIns(4,5)P_2_ and PIP_3_ are dynamically regulated by Hedgehog signalling. Hedgehog pathway stimulation of MEFs using the small molecule SMO activator SAG (SMO agonist) decreases transition zone PtdIns(4,5)P_2_ levels and increases PIP_3_ [6], possibly as PtdIns(4,5)P_2_ is phosphorylated at the 3-position by an unknown class I PI3K.

INPP5E localises to the transition zone in a subset of cells and tightly controls the local levels of the transition zone phosphoinositides, essential for transition zone barrier function and thereby Hedgehog signalling [6]. In SAG-treated *Inpp5e*-null MEFs the transition zone levels of both PtdIns(4,5)P_2_ and PIP_3_ are elevated relative to SAG-treated wild-type MEFs (Figure 6) [6]. Under these conditions of aberrantly high transition zone PtdIns(4,5)P_2_/PIP_3_ in SAG-treated *Inpp5e*-null MEFs, specific defects in transition zone composition were uncovered with functional implications that could explain aspects of the *INPP5E* mutant phenotype. This includes a failure of the transition zone proteins MKS1, TCTN1, TMEM231 and B9D1 to correctly localise to this compartment [6]. Another group of proteins that are affected by *Inpp5e* deletion are septins, a family that form diffusion barriers in cells in general, including at the cilia base where they are proposed to contribute to localisation of transition zone components and retention of receptors in the cilium [19,143,144]. SEPT2 is a septin family member that localises to the axoneme and cilia base [144] and its levels were reduced at the ciliary base in SAG-treated *Inpp5e* KO MEFs [6]. Many transition zone proteins contain C2 and B9 domains [20] and SEPT2 interacts with phosphoinositides including PtdIns(4,5)P_2_ and PIP_3_ via a polybasic region [145], suggesting SEPT2 and transition zone protein mislocalisation in SAG-treated *Inpp5e*-null MEFs may directly relate to the aberrant transition zone phosphoinositide composition.

The reduced recruitment of the ciliary receptors SMO, polycystin II and HTR6 to SAG-treated *Inpp5e*-null cilia is reflective of defective transition zone barrier function [6]. However, given that the non-ciliary protein CEACAM-1 was still excluded from the axoneme, mechanisms governing ciliary entry appear to remain intact. To assess cilia retention, fluorescence recovery after photobleaching of GFP-tagged SMO was examined in *Inpp5e*-null cells. An increased turnover of ciliary GFP-SMO under these conditions revealed that the ciliary retention mechanisms of the transition zone are indeed compromised [6].

An inability of SMO to accumulate in the SAG-stimulated *Inpp5e*-null cilium could explain the abnormal localisation of downstream Hedgehog pathway components in these cells. Firstly, GLI2 concentrates at the cilia tip for SMO-dependent activation [38], but this response was blunted in *Inpp5e* KO cells [6]. Secondly, SMO localisation to the cilium promotes GPR161 cilia exit [146], therefore reduced ciliary SMO could be the cause of increased ciliary GRP161 levels in *Inpp5e*-null cells [4–6]. Therefore, defective ciliary retention mechanisms and the inability of SMO to accumulate in the cilia membrane resulting from abnormal transition zone phosphoinositide composition are likely to contribute to the Hedgehog-dependent phenotypes and repressed Hedgehog signalling in *Inpp5e* KO embryos and cells (Figure 6) [4–6].

Similarly, SAG-stimulated ciliary SMO localisation was blunted in Lowe syndrome murine model fibroblasts, which exhibit elevated transition zone PtdIns(4,5)P_2_ levels [95]. OCRL also hydrolyses PIP_3_ but its levels have not been assessed in these cells, therefore disruption of transition zone PtdIns(4,5)P_2_ and PIP_3_ could contribute to SMO mislocalisation in this context.

In *Drosophila* chordotonal neurons, the INPP5E homologue dINPP5E localises to the cilia base most likely the transition zone, in a region surrounded by high PtdIns(4,5)P_2_ levels [8]. Consistent with murine cells [4–6], *dInpp5e* mutation or knockdown leads to increased PtdIns(4,5)P_2_ levels at this site, associated with increased dTULP cilia recruitment and mislocalisation of the receptors Inactive and NOMPC to the proximal end of the cilium [8]. As an alternative approach to elevate transition zone PtdIns(4,5)P_2_, the PtdIns(4)P-5-kinase Skittles was ectopically expressed in these *Drosophila* neurons resulting in a similar phenotype [8]. Notably, inactivating mutation of *Skittles* in the *Drosophila* male germline induced cilia with abnormally elongated transition zones and functional abnormalities such as defective transition zone plasma membrane tethering [147]. Although this study did not assess the effect of *Skittles* mutation on the transition zone PtdIns(4,5)P_2_ levels, a previous study showed ectopic Skittles controls this phosphoinositide pool [8], suggesting that transition zone PtdIns(4,5)P_2_ signals are essential for transition zone maturation and normal ciliogenesis [147].

#### Transition zone PIP_3_ activates a local signalling axis to control cilia assembly/stability

Primary cilia are in a constant dynamic equilibrium between assembly and disassembly [148–151]. In addition to the involvement of INPP5E, OCRL and INPP5B in cilia assembly and stability [7,11,12,29,77,102,107,115,119,132], there is also indirect evidence to implicate class I PI3K signalling in these phenomena.

**Figure 7.**
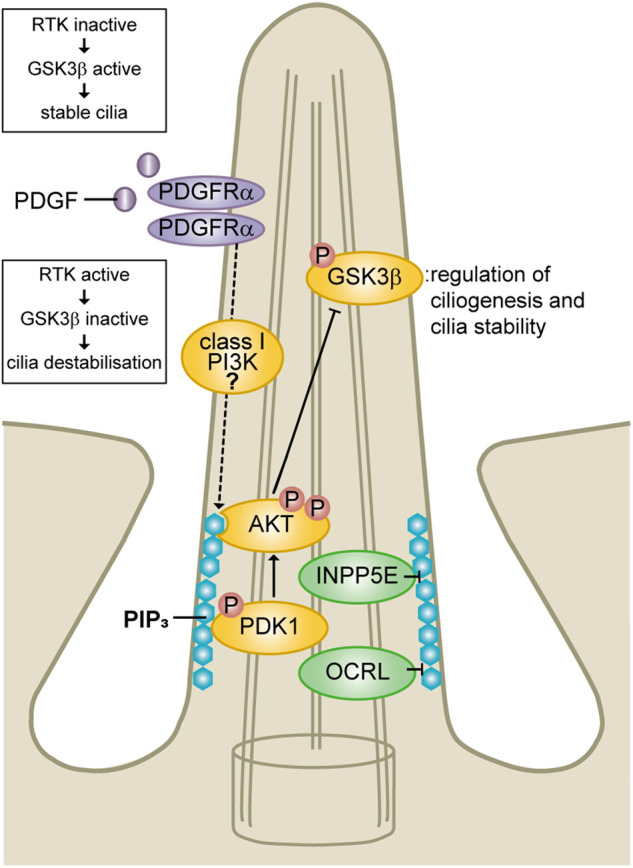
Regulation of cilia stability by transition zone PIP_3_. PIP_3_ localises to the transition zone and its levels increase following stimulation by growth factors possibly via an unknown class I PI3K. Increased transition zone PIP_3_ activates a localised AKT signalling axis which inactivates GSK3β contributing to reduced cilia stability. Local PIP_3_ is negatively regulated by the cilia-localised PI 5-phosphatases INPP5E and OCRL.

Class I PI3Ks phosphorylate PtdIns(4,5)P_2_ to generate PIP_3_ at the plasma membrane in response to stimulation of RTKs or GPCRs [55]. PIP_3_ and its dephosphorylation product PtdIns(3,4)P_2_ act as second messengers by recruiting and activating PH domain-containing proteins [63]. The serine/threonine kinase AKT is one of the most well studied PIP_3_/PtdIns(3,4)P_2_ effector proteins, which, following PIP_3_/PtdIns(3,4)P_2_-driven recruitment to the plasma membrane, becomes fully activated by PDK1- and mTORC2-mediated phosphorylation [152]. The following observations suggest ciliary PIP_3_ and class I PI3Ks may contribute to the mechanisms controlling cilia dynamics (Figure 7).

1. Depletion of the PTEN phosphatase which converts PIP_3_ to PtdIns(4,5)P_2_ and directly opposes PI3K signalling [153], reduces cilia stability, although this is proposed to at least in part rely on the protein phosphatase activity of PTEN [154].
2. Activation of RTKs such as PDGFR upstream of class I PI3K induces cilia disassembly, which is rescued by inhibition of PI3K or AKT [7,11,155–157].
3. Overexpression of wild-type or constitutively active AKT suppresses cilia assembly [7]. AKT and its downstream target GSK3β localise to primary cilia [66,158]. In its non-phosphorylated active form, GSK3β promotes cilia assembly and stability [159,160] and is inhibited via AKT-mediated phosphorylation [161].
4. Providing more direct evidence that ciliary PIP_3_ regulates the cilia assembly/disassembly balance, this lipid localises to the transition zone, with its levels increasing following disassembly-inducing growth factor stimulation [6,7].
5. Other evidence for a role of ciliary PIP_3_ in cilia assembly/disassembly comes from a cilia- and Hedgehog-dependent form of the brain tumour medulloblastoma [162,163]. *Inpp5e*-null Hedgehog-activated murine medulloblastoma cells exhibit reduced cilia assembly *in vivo* and *in vitro*, with increased transition zone PIP_3_ levels in cells isolated from this model. This is associated with increased localisation of activated phospho(p)S241-PDK1 and pT308-AKT to the cilia base and elevated phosphorylated/inhibited pS9-GSK3β recruitment to the axoneme [7]. However, no changes in the pathway were observed at the whole cell level [7]. Although INPP5E-mediated PIP_3_ hydrolysis produces PtdIns(3,4)P_2_ which is also capable of recruiting AKT, many studies have shown loss of 5-phosphatase family members including INPP5E activate AKT signalling via PIP_3_ accumulation [103,164–172]. The number of ciliated *Inpp5e*-null Hedgehog-dependent medulloblastoma cells was rescued by pan-PI3K inhibition and wild-type (but not catalytically inactive) INPP5E [7]. Pan-PI3K inhibition also rescued the increased ciliary PIP_3_ levels in these *Inpp5e* KO cells, collectively suggesting cilia-specific PIP_3_ signalling to AKT and thereby GSK3β inhibition may be an important regulatory mechanism controlling cilia dynamics in medulloblastoma [7].
6. Joubert syndrome and Lowe syndrome patient cells with *INPP5E* and *OCRL* mutations, some of which have been shown to reduce PIP_3_ 5-phosphatase activity, exhibit reduced cilia stability and assembly respectively [12,77,115], suggesting the ciliary PIP_3_/AKT/GSK3β axis may play a role in these diseases, but this is yet to be directly tested.
7. mTORC1 signals downstream of PIP_3_/AKT and has been suggested to positively and negatively regulate ciliogenesis indirectly via modulation of autophagy [173]. However, multiple signalling pathways feed into mTORC1 and it has not been shown that mTORC1 activity is regulated by ciliary phosphoinositides.

#### Transition zone PtdIns(3,4)P_2_ function is relatively unexplored

PtdIns(3,4)P_2_ localises to the transition zone, however, its function, significance and metabolism at this site are largely unknown [7]. Transition zone PtdIns(3,4)P_2_ may be produced by 3-position phosphorylation of PtdIns(4)P by a cilia-associated PI3K such as PI3K-C2α or potentially via 5-phosphatase hydrolysis of PIP_3_ [7,9]. Indeed a subtle reduction in PtdIns(3,4)P_2_ transition zone intensity was observed following *Inpp5e* deletion in medulloblastoma cells [7].

The BBSome is a multi-protein complex that forms a coat on membranes to sort and facilitate entry of membrane proteins into the ciliary membrane [174]. In the cilia model organism *Trypanosoma brucei* the BBSome localises to the transition zone [175]. In humans, mutations in BBSome components cause the ciliopathy BBS characterised by obesity, blindness, polydactyly and mental retardation [176]. The BBSome is recruited onto membranes by the GTPase ARL6 (also known as BBS3) [174]. The BBSome component BBS5 is also thought to bind directly to membranes [133]. BBS5 contains two phosphoinositide binding PH domains that were shown in lipid overlay studies to preferentially bind PtdIns(3)P, but also interact with PtdIns(3,4)P_2_, PtdIns(3,5)P_2_, PtdIns(4)P and PtdIns(5)P [133]. In a slightly more physiologically relevant assay, the recruitment of the BBSome onto liposomes by ARL6 was enhanced by the inclusion of phosphoinositides in the liposomes and most dramatically by PtdIns(3,4)P_2_ [174]. Given that PtdIns(3,4)P_2_ and the BBSome localise to the transition zone [7,175], these data suggest the BBSome may be recruited to the transition zone via a BBS5-PtdIns(3,4)P_2_ interaction *en route* to deliver its cargo membrane proteins into the ciliary membrane. The difference in phosphoinositide binding specificity between liposome and lipid overlay assays may result from a change in lipid binding specificity when lipids are presented immobilised on a membrane *versus* in a lipid bilayer [174].

However, recent structural studies have raised doubts about this model. Firstly, the phosphoinositide binding sites are not conserved in the BBS5 PH domains and the putative phosphoinositide binding pockets are occluded by another BBSome component in the 3D-structure of the complex [177]. Therefore, if these BBS5 sites were to bind PtdIns(3,4)P_2_ or any other phosphoinositide, the complex would need to undergo an unknown conformational change to expose the critical residues [177]. Secondly, a purified BBSome lacking BBS5 (containing only BBS4, BBS8, BBS9 and BBS18) bound the same phosphoinositides [PtdIns(3)P, PtdIns(3,5)P_2_, PtdIns(4,5)P_2_, PtdIns(5)P and PtdIns(3,4)P_2_] in lipid overlay assays as BBS5 alone and the BBSome containing BBS5, indicating that at least *in vitro* other BBSome components are also capable of phosphoinositide binding [178].

#### Unanswered questions

One of the most important questions in the context of the ciliary transition zone is which enzyme produces PIP_3_ and PtdIns(3,4)P_2_ in this location. It seems reasonable to hypothesise that (a) class I PI3K(s) phosphorylates PtdIns(4,5)P_2_ at the transition zone for local PIP_3_ production. However, all studies to date have used pan-PI3K inhibitors [7,11]. Moreover, it is unknown whether class I PI3Ks enzymes localise to cilia or whether the lipid is transported from elsewhere. Technical limitations make it difficult to determine the precise subcellular localisation of endogenous and recombinant class I PI3Ks [179]. Furthermore, it is possible that different class I PI3K isoforms contribute to the transition zone PIP_3_ pool under basal and growth factor stimulated conditions. Conversely, the contribution of cilia biology to the function of class I PI3K(s) remain unknown.

PtdIns(3,4)P_2_ is produced by both class II PI3K phosphorylation of PtdIns(4)P and by 5-phosphataste hydrolysis of PIP_3_ and it is unknown which of these is the major pathway required for transition zone PtdIns(3,4)P_2_ production.

It is also unclear precisely how PtdIns(4,5)P_2_ and PIP_3_ function to regulate transition zone composition and how disease-related changes in the levels of these lipids lead to mislocalisation of transition zone components and diffusion barrier dysfunction. Similar to *Inpp5e*-null cells, fibroblasts from the Lowe syndrome mouse model exhibit reduced SAG-stimulated SMO ciliary localisation [95]. The functional significance of these observations is unclear given that Lowe syndrome does not present with typical Hedgehog loss-of-function phenotypes and that *OCRL* mutant or KO cells have not been shown to exhibit a defective Hedgehog transcriptional response [105,106].

## Conclusions and perspectives

Significant progress has been made in understanding the phosphoinositide composition of the primary cilium and the function of these lipids at cilia. It is becoming increasingly clear that ciliary phosphoinositides are critical regulators of multiple processes covering the entire cilia life cycle, from the initiation of ciliogenesis and ciliary trafficking to disassembly. An emerging theme, also common to non-ciliary phosphoinositides, is that the same lipid exhibits distinct functions at different ciliary subdomains, likely depending on the local phosphoinositide-binding effectors.

All studies to date have utilised phosphoinositide-binding biosensors or monoclonal antibodies for lipid detection at the cilium [4–9,29]. As detailed in Box 1, these techniques have limitations and some caution needs to be exercised in interpreting the reported subcellular localisation of these phosphoinositides and a combination of detection methods and specificity validation is required.

Despite the recent advances described here, many unanswered questions remain regarding ciliary phosphoinositide function and metabolism. Key outstanding questions include addressing how the cilium maintains its unique phosphoinositide complement, is this via the action of local lipid kinases and phosphatases or is it largely controlled by diffusion barriers? Are all phosphoinositides produced locally at cilia or trafficked from an endocytic compartment or the plasma membrane into the ciliary membrane and if they are produced locally, what is the source of non-phosphorylated PtdIns?

Ultimately the most important question is how to harness the growing understanding of ciliary phosphoinositides to design treatments for ciliopathy syndromes caused by defective phosphoinositide metabolism at cilia. The high druggability of the enzymes regulating phosphoinositide metabolism, currently mainly exploited in the context of cancer and immune regulation, may also offer opportunities to alleviate suffering in ciliopathy syndromes.

## Open Access

Open access for this article was enabled by the participation of University College London in an all-inclusive *Read & Publish* pilot with Portland Press and the Biochemical Society under a transformative agreement with JISC.

## Abbreviations

BBS: Bardet–Biedl syndrome
E10.5: embryonic day 10.5
GCP: granule cell progenitor
GEF: guanine nucleotide exchange factor
GPCR: G protein-coupled receptor
IFT: intraflagellar transport
KO: knockout
MEF: mouse embryonic fibroblast
MORM: mental retardation, obesity, congenital retinal dystrophy and micropenis in males
p: Phospho
PDGFR: Platelet-Derived Growth Factor-Receptor
PH: Pleckstrin Homology
PI3K: phosphoinositide 3-kinase
PIP_3_: Phosphatidylinositol (3,4,5)-trisphosphate
PIPKIγ: Type Iγ PtdIns(4)P 5-kinase
PTCH1: Patched 1
PtdIns: phosphatidylinositol
PtdIns(3)P: Phosphatidylinositol 3-phosphate
PtdIns(3,4)P_2_: Phosphatidylinositol 3,4-bisphosphate
PtdIns(3,4,5)P_3_: Phosphatidylinositol (3,4,5)-trisphosphate
PtdIns(3,5)P_2_: Phosphatidylinositol 3,5-bisphosphate
PtdIns(4)P: Phosphatidylinositol 4-phosphate
PtdIns(4,5)P_2_: Phosphatidylinositol 4,5-bisphosphate
RTK: receptor tyrosine kinase
SAG: SMO agonist
SMO: Smoothened
SUFU: Suppressor of fused
TTBK2: tau-tubulin kinase-2
TULP3: Tubby-like protein 3

Box 1: Influence of phosphoinositide detection methods on apparent cilia localisation PtdIns(4,5)P_2_ is the best studied ciliary phosphoinositide, with its cilia localisation reported to vary slightly between studies, ranging from the transition zone, proximal end, cilia base and one study showing localisation to the proximal end of the cilium in *Inpp5e*-null cells [4–8,95,126]. These differences in reported distributions likely result from the different phosphoinositide detection methods that were used in each study. Expression of fluorescently-labelled phosphoinositide binding biosensors, such as the PtdIns(4,5)P_2_-binding PH domain of phospholipase Cδ, have been a mainstay of phosphoinositide detection for many years [180]. These tools are limited however by the potential off-target effects resulting from lipid sequestration by directly competing with endogenous lipid binding proteins and preventing the access of lipid-metabolising enzymes [180]. Furthermore, the biosensors may not report an accurate localisation if their access to the lipid is restricted by a diffusion barrier or entrapment by proximal lipid pools [181]. Immunofluorescence using phosphoinositide specific monoclonal antibodies can overcome these issues, but the staining pattern observed is highly dependent on the cell permeabilisation method used, as lipids are not cross-linked during fixation and are liable to extraction [182]. PtdIns(4,5)P_2_ was observed at the transition zone using either digitonin-permeabilisation optimised for detection of intracellular membrane phosphoinositides or methanol permeabilisation [6,7,182]. In contrast, Triton X-100 permeabilises endomembranes [183] and studies using this permeabilisation method detected ciliary membrane PtdIns(4,5)P_2_ only following lipid 5-phosphatase depletion [4,95]. Therefore, although a consensus seems to exist supporting PtdIns(4,5)P_2_-enrichment at the cilia base, further studies using multiple methods of validation, co-localisation with cilia sub-compartment markers, superresolution and live cell imaging will be required to precisely elucidate its localisation. Notably, many of the protocols used to detect ciliary phosphoinositides are incompatible with plasma membrane phosphoinositide staining, meaning that even though PtdIns(4,5)P_2_ is abundant at the plasma membrane, studies directly comparing ciliary and plasma membrane phosphoinositide pools have not been possible [4,6,7,95,182].
